# Authentication of M14 melanoma cell line proves misidentification of MDA‐MB‐435 breast cancer cell line

**DOI:** 10.1002/ijc.31067

**Published:** 2017-10-10

**Authors:** Christopher Korch, Erin M. Hall, Wilhelm G. Dirks, Margaret Ewing, Mark Faries, Marileila Varella‐Garcia, Steven Robinson, Douglas Storts, Jacqueline A. Turner, Ying Wang, Edward C. Burnett, Lyn Healy, Douglas Kniss, Richard M. Neve, Raymond W. Nims, Yvonne A. Reid, William A. Robinson, Amanda Capes‐Davis

**Affiliations:** ^1^ International Cell Line Authentication Committee (ICLAC); ^2^ Division of Medical Oncology University of Colorado Anschutz Medical Campus Aurora CO; ^3^ Genetica Cell Line Testing – a LabCorp brand Burlington NC; ^4^ Leibniz‐Institute DSMZ – Deutsche Sammlung von Mikroorganismen und Zellkulturen Braunschweig Germany; ^5^ Promega Corporation Madison WI; ^6^ John Wayne Cancer Institute Santa Monica CA; ^7^ Culture Collections Public Health England Porton Down United Kingdom; ^8^ Biological Research Facility The Francis Crick Institute London United Kingdom; ^9^ Departments of Obstetrics and Gynecology and Biomedical Engineering The Ohio State University Columbus OH; ^10^ Gilead Sciences Inc Foster City CA; ^11^ RMC Pharmaceutical Solutions, Inc. Longmont CO; ^12^ American Type Culture Collection (ATCC) Manassas VA; ^13^ CellBank Australia, Children's Medical Research Institute, The University of Sydney Westmead NSW Australia

**Keywords:** authentication, cross‐contamination, human cell lines, misidentification, STR profiling

## Abstract

A variety of analytical approaches have indicated that melanoma cell line UCLA‐SO‐M14 (M14) and breast carcinoma cell line MDA‐MB‐435 originate from a common donor. This indicates that at some point in the past, one of these cell lines became misidentified, meaning that it ceased to correspond to the reported donor and instead became falsely identified (through cross‐contamination or other means) as a cell line from a different donor. Initial studies concluded that MDA‐MB‐435 was the misidentified cell line and M14 was the authentic cell line, although contradictory evidence has been published, resulting in further confusion. To address this question, we obtained early samples of the melanoma cell line (M14), a lymphoblastoid cell line from the same donor (ML14), and donor serum preserved at the originator's institution. M14 samples were cryopreserved in December 1975, before MDA‐MB‐435 cells were established in culture. Through a series of molecular characterizations, including short tandem repeat (STR) profiling and cytogenetic analysis, we demonstrated that later samples of M14 and MDA‐MB‐435 correspond to samples of M14 frozen in 1975, to the lymphoblastoid cell line ML14, and to the melanoma donor's STR profile, sex and blood type. This work demonstrates conclusively that M14 is the authentic cell line and MDA‐MB‐435 is misidentified. With clear provenance information and authentication testing of early samples, it is possible to resolve debates regarding the origins of problematic cell lines that are widely used in cancer research.

## Abstract 2

**What's new?**

A variety of analytical approaches have indicated that melanoma cell line M14 and breast carcinoma cell line MDA‐MB‐435 originate from a common donor, but there is ongoing debate regarding which is the misidentified cell line. Here, authentication testing of M14 from 1975 (prior to the establishment of MDA‐MB‐435), with comparison to donor serum and lymphoblastoid cell line ML14, shows that M14 is the authentic cell line and MDA‐MB‐435 is a misidentified derivative. With clear provenance information and authentication testing of early samples, debates regarding the origins of problematic cell lines that are widely used in cancer research can be resolved.

## MDA‐MB‐435 Breast and M14 Melanoma Cell Lines Share a Common Origin

**Figure 1:**
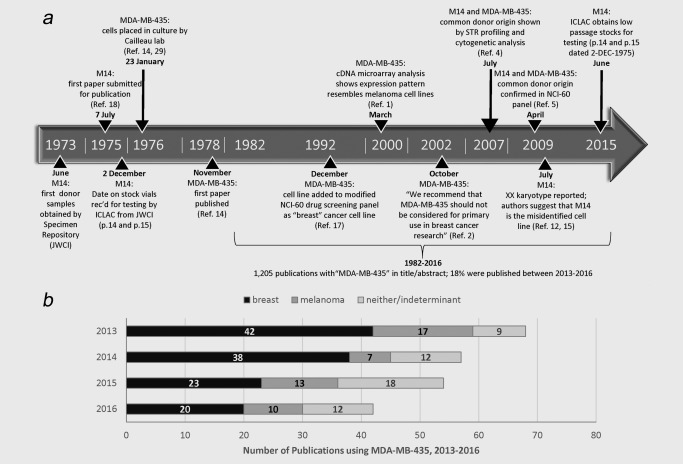
M14 and MDA‐MB‐435 timeline and recent publications. (*a*) Timeline of events in the establishment and analysis of M14 and MDA‐MB‐435. Ref = citation from Reference list; JWCI = John Wayne Cancer Institute. (*b*) Usage of MDA‐MB‐435 in journal publications between January 2013 and December 2016 (looking at date of hard copy publication). A search was conducted to look for “MDA‐MB‐435” in the title or abstract, and available text was examined to classify usage as “breast” or “melanoma.” If authors described MDA‐MB‐435 more broadly as a cancer cell line, or lack of access to the full text meant that usage could not be determined, usage was classified as “neither/indeterminant.”

The authenticity of two cell lines, MDA‐MB‐435 and UCLA‐SO‐M14 (commonly referred to as M14), has been debated in the scientific literature over many years (timeline: see Fig. 1 *a*). Questions were first raised regarding the tissue origin of MDA‐MB‐435 in 2000, when cDNA microarray analysis of the NCI‐60 panel showed that the expression pattern of the claimed breast carcinoma cells closely resembled patterns seen in melanoma cell lines.^1^ Similar results were reported by other laboratories.^2^ Subsequent analysis of multiple samples of MDA‐MB‐435 showed that cell stocks in use at different laboratories around the world shared common expression patterns associated with melanoma.^3^

Numerous analytical approaches (karyotyping, comparative genomic hybridization, microsatellite polymorphism analysis, STR analysis, single nucleotide polymorphism (SNP) analysis and bioinformatics analysis of gene expression) have demonstrated that the MDA‐MB‐435 cell line shares a common origin with the M14 melanoma cell line.^4^, ^5^ These studies have concluded that MDA‐MB‐435 is misidentified and is in fact a derivative of the M14 melanoma cell line.^4^, ^6^, ^7^, ^8^

This conclusion, however, has been debated on the basis of phenotypic evidence. MDA‐MB‐435 can express breast‐specific and epithelial‐specific markers, leading to the conclusion that it is derived from breast carcinoma and that expression of melanocyte‐specific markers may be caused by lineage infidelity,^9^, ^10^ possibly as a normal property of freshly excised mammary tumor tissue.^11^

The provenance of M14 and MDA‐MB‐435 was reviewed in a 2009 letter, resulting in further concerns regarding the authenticity of M14.^12^ The letter observed that the sex of origin recorded for the two cell lines is different. M14 was derived from a male and MDA‐MB‐435 from a female donor.^13^, ^14^ Since cytogenetic analysis had shown that MDA‐MB‐435 has an XX karyotype, the author postulated that M14 may be the misidentified cell line.^12^, ^15^ However, not all evidence supports this conclusion. Y‐specific markers were recently detected in MDA‐MB‐435 using the SNP Trace™ System, which includes three X‐specific and three Y‐specific SNP loci.^16^

## MDA‐MB‐435 Continues to be Widely Used as a Breast Carcinoma Model

MDA‐MB‐435 and derivatives such as MDA‐MB‐435S (ATCC HTB‐129, a spindle‐shaped variant of the parental cell line) continue to be used extensively and described as “breast carcinoma” in the scientific literature.^6^, ^8^ For example, in December 1992, MDA‐MB‐435 was added to the NCI‐60 cell line panel for use in anticancer drug screening, where it was initially described as a “breast” cancer cell line.^17^ STR profiling of the NCI‐60 panel later confirmed the shared donor origin of these cells with M14.^5^

A recent search of PubMed using the search term “MDA‐MB‐435” shows that the cell line was referred to in the title or abstract of 1,205 publications between 1982 and 2016. MDA‐MB‐435 was described as a breast cell line in 56% (123/221) of recent publications between 2013 and 2016 (Fig. 1 *b*). Many laboratories have used MDA‐MB‐435 as a breast cancer model, because of its rapid growth and ability to metastasize in nude mice, to develop treatments for breast cancer, or to help understand how breast cancers metastasize.^7^

Publications that cast doubt on the authenticity of M14 are frequently cited. For example, the letter referring to the sex of origin of M14 and MDA‐MB‐435^12^ has been cited 159 times (Web of Science, July 25, 2017). It seems likely that ongoing usage of both cell lines in the scientific literature has been influenced by the lack of resolution of the debate regarding the authenticity of the cell lines. Therefore, it is important to explore the origins of M14 and MDA‐MB‐435 to see if this ongoing debate can now be resolved to the satisfaction of the research community.

## Material and Methods

### Cell lines and culture conditions

The International Cell Line Authentication Committee (ICLAC) obtained frozen samples corresponding to the M14 donor (serum and cell lines), MDA‐MB‐435 and derivative MDA‐MB‐435S. Samples were used for DNA extraction or culture.

Samples from the donor of M14 were requested from the John Wayne Cancer Institute (JWCI) Sample Repository. The Sample Repository informed us that serum and cell lines, but not tissue samples, were maintained from this donor. Samples from the M14 donor were first deposited in June 1973. The Specimen Repository provided the following samples for testing:

1. One tube of donor serum, dated December 21, 1973.
2. Two vials of M14 (melanoma^18^), labeled as passage 14 and 15, both dated December 2, 1975. The M14 passage 15 vial was thawed and approximately three quarters of the sample was used for culture, with the remainder used for DNA extraction. The M14 passage 14 vial was thawed and used for DNA extraction to check that STR profiling data were reproducible across both samples.
3. One vial of ML14 (synonymous lymphoblastoid cell line^19^), dated January 9, 1998 with no passage number recorded. The ML14 vial was thawed and used for culture and DNA extraction.

Requests for M14 or ML14 cells may be made to the JWCI Specimen Repository.

M14 (adherent culture) and ML14 cells (suspension culture) were grown at 37°C and 5% CO_2_ in RPMI 1640 (ThermoFisher Scientific, New York, NY; catalogue number 10–04‐CV) with 10% fetal bovine serum (FBS) and 1% penicillin and streptomycin. Mycoplasma testing was performed using the e‐Myco *plus* Mycoplasma PCR Detection kit (Bulldog Bio, Portsmouth, NH; catalogue number 25234), a PCR‐based assay containing internal and sample controls. Mycoplasma were not detected in M14 or ML14 cultures.

A sample of MDA‐MB‐435 was requested from the authors of an earlier study that demonstrated breast‐differentiation specific markers in MDA‐MB‐435 cells.^9^ A sample was provided for testing, dated 2000 and labeled “JP.” A sample of MDA‐MB‐435S (HTB‐129) was also requested from ATCC. In response, ATCC provided cells (lot 60235534, passage 345), genomic DNA (lot 59887026, passage 345) and historical deposit information. The historical deposit record noted that MDA‐MB‐435S (passage 233) was deposited on April 6, 1982 by Dr Relda Cailleau at EG&G Mason Research Institute, and later transferred to ATCC.

### Immunostaining

M14 cells at passage 16 (one passage after thawing) were plated on a slide and grown to 80% confluence. Slides were fixed with formalin and immunohistochemistry performed using the Melanoma Triple Cocktail stain (Ventana Medical Systems, Tucson, AZ; catalogue number 790–4,677), a mixture of three mouse monoclonal antibodies directed against melanosome (HMB45), MART‐1/Melan A (A103) and tyrosinase (T311).^20^ Slides were processed using the Benchmark XT® automated stainer as described in the Supporting Information Methods.

### Genomic DNA and DNA extraction

DNA extraction for STR analysis from cultured cells was performed using the Quick‐gDNA MiniPrep kit (Zymo Research Corporation, Valencia, CA; catalogue number D3024). DNA extraction for ABO sequencing was performed using the DNeasy Blood & Tissue kit, using the protocol for Total DNA from Animal Blood or Cells (Qiagen, Valencia, CA; catalogue number 69506). Pre‐treatment was performed using 25 µL RNase A (100 mg/ml; catalogue number 19101) with incubation at 56°C for 10 min prior to purification. DNA was eluted in 10 mM Tris‐HCl, pH 8 and its concentration and quality assessed by gel electrophoresis and spectrophotometric measurement.

DNA extraction from serum was performed using a different method that was previously shown to be effective for serum samples. A detailed procedure and references are supplied in the Supporting Information Methods section.

### ABO sequencing

To examine the ABO gene locus, primers were designed to amplify exon 6 of the human ABO locus. This region includes nucleotide 261; deletion of this nucleotide occurs in individuals that carry the O allele, resulting in a frameshift mutation.^21^ Primer sequences were as follows: forward, GCAGAAGCTGAGTGGAGTTT; and reverse, TAACCCAATGGTGGTGTTCTG.

DNA from the M14 cell line was amplified using primers at a final concentration of 0.4 µM and 300 ng of genomic DNA. Cycle conditions were as follows: initial hold at 95°C, 2 min; followed by 25 cycles at 94°C, 30 sec; 64°C, 1 min; 72°C, 30 sec; followed by 15 cycles at 94°C, 30 sec; 53°C, 1 min; 72°C, 1 min; followed by 10 min at 72°C, with final hold at 10°C. The PCR products were treated with ExoSAP‐IT (Affymetrix USB, Cleveland, OH; catalogue number 78201) and submitted for DNA sequencing. Amplicons were sequenced using a 3130 Genetic Analyzer with a BigDye® Terminator v3.1 Cycle sequencing Kit (v3.1; catalogue number 4337455) and using POP7 (Applied Biosystems, ThermoFisher Scientific).

### STR and SNP genotyping

STR profiling to examine the core set of loci used for cell line authentication^22^ was performed using the AmpFLSTR® Identifiler^®^ kit, with a reduced volume modification of the manufacturer's instructions (Applied Biosystems, ThermoFisher Scientific; catalogue number 4322288). Identifiler^®^ data were obtained using an ABI 3730 genetic analyzer and analyzed using GeneMapper 4.0 software (Applied Biosystems, ThermoFisher Scientific). Percent match comparisons were made using the Tanabe algorithm^23^, ^24^; a step‐by‐step workflow for profile comparison is set out in the Supporting Information Methods.

X‐Chromosome‐specific STR loci were analyzed using a DXS multiplex PCR, developed at the DSMZ. A detailed procedure for the DXS multiplex PCR is set out in the Supporting Information Methods. Control samples were included for comparison from the male cell line CAKI‐2, and the female cell line A‐204 (Supporting Information Table S3).

Y‐Chromosome‐specific STR loci and autosomal STR loci were analyzed using PowerPlex^®^ Y23 and PowerPlex^®^ Fusion 6C Systems (Promega, Madison, WI), in accordance with the manufacturer's instructions.^25^, ^26^ Data were analyzed using GeneMapper ID‐X v1.4.

SNP analysis was performed using the Fluidigm SNP Trace™ Panel (Fluidigm, South San Francisco, CA; catalogue number 100–6,280) as previously reported.^16^ Percent match comparisons used the same workflow employed for STR profiling. Allele calls for sex‐specific SNP loci were confirmed by direct PCR amplification and sequencing of the relevant loci as described in the Supporting Information Methods.

Locations of all Y‐STR and Y‐SNP loci evaluated in the current study are shown in Supporting Information Figure S1.

### G‐banding and fluorescence *in situ* hybridization (FISH) analyses

Cells were harvested after incubation with Colcemid (0.05 µg/mL) for 2 hrs. Cells were detached using trypsin‐EDTA, treated with KCl hypotonic solution, fixed, and dropped onto a pre‐cleaned slide. FISH was performed with the Vysis CEP X SpectrumOrange/Yq12 SpectrumGreen probe set (Abbott Molecular, Des Plaines, IL; catalogue number 07J22–050) using standard methodology, as previously described.^27^ The FISH probe set included a CEP X probe recognizing homology to the Xp11‐q11 centromeric region (rich in satellite I DNA) and a Yq12 probe recognizing homology to the heterochromatic Yq12 region (rich in satellite III DNA).

G‐banding was performed also with standard methodology^28^ using trypsin and Leishman's staining (GTL). Sequential G‐banding and FISH were carried out by completely de‐staining the GTL‐stained slide after banding analyses through incubations in ethanol and 3:1 methanol: acetic acid fixative for 4 hrs and then performing FISH as described. Chromatin was counterstained in 0.3 µg/mL DAPI in mounting medium (Vector Laboratories, Burlingame, CA; catalogue numbers H‐1000 and H‐1200).

## Results

### Cell line provenance shows that M14 was established before MDA‐MB‐435

The International Cell Line Authentication Committee (ICLAC) maintains a list of known misidentified cell lines based on testing and known provenance (http://iclac.org/databases/cross-contaminations/). The debate, and ensuing confusion surrounding M14 and MDA‐MB‐435, prompted the committee to review the scientific literature in an attempt to verify the provenance of these two cell lines.

M14 was reported to be established at the University of California Los Angeles (UCLA) from a 33‐year‐old patient with metastatic melanoma; tissue was removed from an amelanotic lesion on the right buttock. A specific date was not recorded in the literature for commencement of M14 culture. The first journal article to describe the M14 cell line was submitted for publication on July 7, 1975.^18^ The donor was reported to be a male with blood type O.^13^

Further review of M14 publications showed that at least two other synonymous cell lines were established from the same donor. ML14 was cultured from the donor's lymphoblasts and immortalized by transfection with EBV; MF14/SV40 was cultured from skin cells and immortalized with SV40 virus.^19^ In the 1970s, the originating laboratory performed authentication testing on synonymous cell lines M14, ML14 and MF14/SV40 using HLA analysis. Results were consistent with a shared origin from the same donor.^19^

MDA‐MB‐435 was reported to be established at the MD Anderson Hospital and Tumor Institute from a 31‐year‐old female patient with metastatic breast carcinoma and were placed into culture on January 23, 1976.^14^, ^29^ Isoenzyme analysis was performed to exclude HeLa contamination^14^; other donor characteristics, such as blood type, were not reported.

Based on our literature review of provenance, we concluded that M14 was in culture for at least 6 months before MDA‐MB‐435 was established (Fig. 1 *a*). Although authentication testing was performed in the 1970s using HLA analysis, showing that M14 corresponded to two synonymous cell lines,^19^ our literature analysis did not exclude the possibility that M14 became misidentified after January 1976 when MDA‐MB‐435 was placed into culture. We therefore attempted to contact the originators of both cell lines or their institutions, to ask for further information and samples for testing. In the case of MDA‐MB‐435, additional information about the patient (*e.g*., blood type) was not available from the MD Anderson Cancer Center.

The originator of M14, Donald Morton, was instrumental in establishing a Specimen Repository at JWCI.^30^ We therefore approached JWCI, which confirmed that M14 and other samples from this donor were still held by this Repository. Their records indicate that M14 was established from a 33‐year‐old male who was diagnosed with primary melanoma in mid‐1971. The primary tumor was an ulcerated lesion (thickness unknown) located on the patient's upper back. Stage III disease was diagnosed four months later and Stage IV disease in 1972. The patient was first seen by Dr. Morton's group in 1973 and samples deposited in the Specimen Repository from June 1973. The sample used to establish M14 was obtained from an amelanotic metastatic lesion removed from the patient's right buttock. The patient received multiple courses of chemotherapy including fluorouracil, doxorubicin, cyclophosphamide and methotrexate; he died from metastatic melanoma in late 1974.

### M14 cells from 1975 express melanoma markers and are blood type O

**Figure 2:**
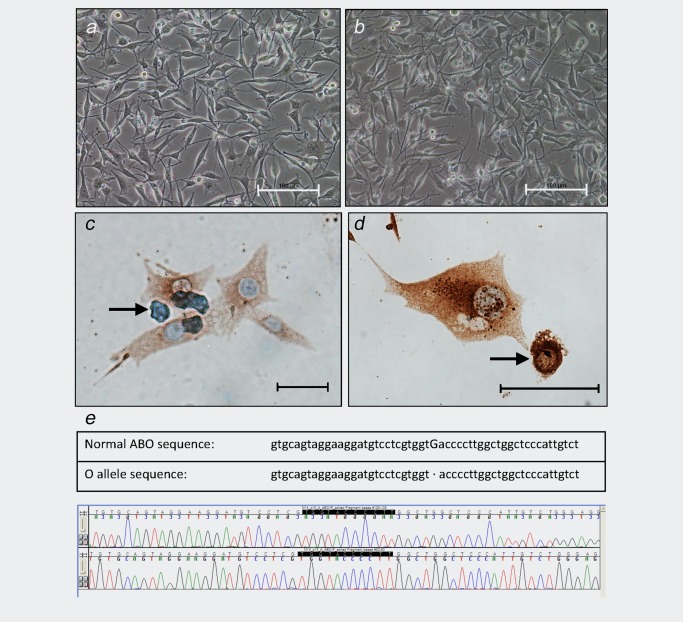
Characterization of M14 using Immunostaining and ABO Analysis. (*a–b*) Representative images of M14 cells in culture. Scale bars, 100 µm. (*c–d*). Immunostaining of M14 cells using a pan‐melanoma antibody cocktail. Antibodies are directed against melanosome (HMB45), MART‐1/Melan A (A103) and tyrosinase (T311). Cells displayed abundant cytoplasm and were multinucleated; some apoptotic cells were noted, as indicated by the arrows. Scale bar in C, 10 µm; scale bar in D, 20 µm. (*e*) ABO sequence demonstrating blood type O. Upper panel, sequence reported previously for A or B alleles and sequence reported for the O allele.^21^ Lower panel, forward and reverse sequence from M14 sample (passage 16, derived from passage 15 from December 2, 1975). Identical sequence results were obtained from ML14 and MDA‐MB‐435S samples (data not shown).

Two vials of M14 (passages 14 and 15) and other samples from this donor were provided by the JWCI Specimen Repository for analysis. The M14 vials were both dated December 2, 1975, 7 weeks before MDA‐MB‐435 cells were first placed into culture (Fig. 1 *a*,^14^). M14 cells were thawed and used for cell culture and DNA extraction. Cells grew well in culture and displayed a spindle‐shaped morphology, similar to publicly available images for MDA‐MB‐435S (Figs. 2 *a* and 2 *b*; ATCC catalogue entry, HTB‐129).

Immunostaining was performed on the M14 cell line (one passage after thawing) to evaluate expression of melanoma‐specific markers. A pan‐melanoma antibody cocktail was used, which is commonly employed for melanoma diagnosis.^20^ M14 cells demonstrated weak to moderate staining (Figs. 2 *c* and 2 *d*), which is consistent with the original sample being from an amelanotic lesion. A melanoma tissue sample, used as the positive control, resulted in strong staining (data not shown).

DNA was extracted from M14 cells (December 1975 samples and one passage after thawing) to evaluate donor characteristics. The donor was reported to be male, a finding that did not concur with karyotypic analysis^12^; donor sex is examined below. The donor was also reported to be blood type O.^13^ To examine blood type, M14 DNA was amplified and sequenced, looking for the single base deletion in exon 6 that determines blood type O.^21^ The sequence from M14 showed that it was homo‐ or hemizygous for a single base pair deletion at nucleotide 261 (Fig. 2 *e*), typically seen in individuals who carry the O allele.^21^ This is consistent with the donor's reported status as blood type O.^13^

M14 DNA was also tested for a BRAF mutation, V600E, which is present in 50–70% of melanoma cases.^31^ BRAF exon 15 was amplified, sequenced and codon 600 was found to be heterozygous for this mutation in M14 (data not shown). The BRAF V600E mutation has been documented in the M14 cell line previously.^15^, ^32^

We concluded that M14 immunostaining, BRAF status and blood type are consistent with the reported tissue type and donor characteristics. However, these characteristics alone were insufficient to conclude that M14 cells were derived from the reported donor. To exclude misidentification, it is important to use authentication testing to compare M14 to other samples from the same donor.

### M14 genotyping corresponds to donor serum, ML14 and MDA‐MB‐435 cells

We asked JWCI if synonymous cell lines^19^ or other donor material were archived in the Sample Repository; a vial of lymphoblast cell line ML14 and an aliquot of donor serum were provided for testing. STR profiles were generated from M14 and ML14 using the Identifiler^®^ kit, which includes 15 autosomal STR loci plus amelogenin for sex determination. Despite containing low amounts of DNA, DNA was successfully extracted from donor serum and an STR profile generated (see Supporting Information Methods for detail).

Serum DNA from the melanoma donor was found to correspond to M14 and ML14 (Table 1). Comparison to donor serum across 16 loci (15 STR loci plus amelogenin) resulted in percent match values of 95% for ML14 and 93% for M14 (Supporting Information Table S1). These results demonstrated a common donor origin for all samples, based on established cell line match criteria.^22^, ^23^, ^24^

**Table 1:** STR analysis of melanoma donor serum and synonymous cell lines ML14, M14 and MDA‐MB‐435S

Cell Line Name	Amelogenin	CSF1PO	D2S1338	D3S1358	D5S818	D7S820	D8S1179	D13S317	D16S539	D18S51	D19S433	D21S11	FGA	TH01	TPOX	vWA
(1) Donor serum	X,Y	11	19,24	14,16	11,12	8,10	13,14	11,12	9,13	13,17	14,15	30	21,26	6,7	8,11	16,18
(2) ML14	X,Y	11	19,24	14,16	11,12	8,10	13,14	11,12,13	13	13,17	14,15	30	21,25,26	6,7	8,11	16,18
(3) M14	X	11	19,24	14,16	11,12	8,10	13	12	9,13	13,17	14,15	30	21	6,7	8,11	16,18
(4) MDA‐MB‐435S	X	11,12	19,24	14	12	8	13,14	12	13	13,17	14,15	30	21	6,7	8,11	16,18

STR loci were amplified using the ABI Identifiler^®^ kit and include the eight core STR loci recommended for human cell line comparison. Sample or profile sources: (1) Serum from donor of M14 and ML14 cell lines, dated December 21, 1973 (source: JWCI); (2) ML14 lymphoblastoid cells, dated January 9, 1998 (source: JWCI); (3) M14 melanoma cells, dated December 2, 1975 (source: JWCI); and (4) MDA‐MB‐435S cells (source: ATCC, HTB‐129D). See also Supporting Information Table S1, which contains additional STR profiles from publications that correspond to the donor serum sample used here as a reference.

Serum DNA from the melanoma donor also corresponded to early samples of MDA‐MB‐435 and MDA‐MB‐435S (Table 1, Supporting Information Table S1). MDA‐MB‐435 cells were dated 2000 and labeled “JP”; the label suggests that cells were obtained from the laboratory of Dr Janet Price.^9^ MDA‐MB‐435S cells were provided by ATCC and were deposited by the originator in 1982. Comparison to donor serum across 16 loci (15 STR loci plus amelogenin) resulted in percent match values of 87% for MDA‐MB‐435 and 85% for MDA‐MB‐435S, consistent with a common donor origin for all samples.^22^, ^23^, ^24^

Looking more broadly, we compiled a dataset of 29 STR profiles from our testing and others' publications, looking for MDA‐MB‐435 and any cell lines corresponding to M14 donor serum (Supporting Information Table S1). STR profiles included an early sample from the MD Anderson Characterized Cell Line Core Facility, tested in 2009. All STR profiles corresponded to serum from the M14 donor; none of the STR profiles from MDA‐MB‐435 exhibited a different donor origin.

Variations were observed at some STR loci across our cell line dataset. Loss of heterozygosity was present in all cultured samples compared to donor serum, which is a common observation for cell line STR profiles.^22^ STR profiles from MDA‐MB‐435 samples displayed increased loss of heterozygosity when compared to donor serum, ML14 and M14 (Supporting Information Table S1). This finding is consistent with MDA‐MB‐435 being a derivative cell line at later passage. In contrast, ML14 displayed a triallelic pattern at the D13S317 and FGA STR loci. A triallelic pattern at some STR loci is not uncommon for cell lines^22^ and has been documented previously in lymphoblastoid cell lines, particularly at the FGA locus.^33^

Sex‐specific markers are of particular interest, considering that M14 is reported to come from a male donor and MDA‐MB‐435 from a female donor. STR profiles typically include amelogenin, using AMELX and AMELY for sex determination.^22^ Donor serum and ML14 carried AMELX and AMELY; M14 and MDA‐MB‐435S carried only AMELX (Table 1).

To assess concordance between test methods, SNP genotyping was performed on M14, ML14 and MDA‐MB‐435S DNA. The SNP Trace™ System was used because it contains sex‐specific SNP loci and published data are available for MDA‐MB‐435.^16^ Serum DNA was not available at the time this analysis was performed, so ML14 DNA was selected as a reference sample (Supporting Information Table S2). Comparison across 96 SNP loci resulted in percent match values of 97% when comparing M14 to ML14, and 91–92% when comparing MDA‐MB‐435S to ML14 (Supporting Information Table S2), confirming a common donor origin for these samples. Loss of heterozygosity was observed and was more evident in MDA‐MB‐435 samples compared to ML14 and M14 (Supporting Information Table S2), consistent with MDA‐MB‐435 being a later passage derivative.

Sex‐specific SNP loci were examined in closer detail. One X‐specific locus, hu103X (rs525869), was called as heterozygous in ML14, M14 and MDA‐MB‐435S (A:G, Supporting Information Table S2). We explored this finding further by performing PCR amplification and sequencing of the hu103X locus. Sequences from ML14, M14 and MDA‐MB‐435S were found to contain a single SNP variant (G, Supporting Information Fig. S2), indicating that the allele call from the SNP Trace™ System was incorrect in this instance. Two other X‐specific SNP loci, hu107X and hu109X, were called as homo‐ or hemizygous and these results were confirmed by sequencing. The SNP Trace™ System also contains three Y‐specific SNP loci, which were previously detected in a sample of MDA‐MB‐435.^16^ In our hands, Y‐specific SNP loci were detected in ML14, but not in M14 or MDA‐MB‐435S. We confirmed this result by using conventional PCR to amplify three Y‐SNP loci used in the SNP Trace™ System and additional autosomal controls. Autosomal loci were detected in ML14, M14 and MDA‐MB‐435S cells; Y‐specific SNP loci were detected only in ML14 (Supporting Information Fig. S3).

We concluded that the STR and SNP profiles previously reported for M14 and MDA‐MB‐435^4^, ^5^, ^16^ correspond to serum from the M14 donor, a synonymous lymphoblastoid cell line, and samples of M14 cryopreserved the year before MDA‐MB‐435 was established. This evidence is sufficient to conclude that MDA‐MB‐435 is misidentified and M14 is authentic. Loss of Y‐specific loci in M14 and MDA‐MB‐435S was also noted, making donor sex difficult to discern in the absence of other donor material. Although loss of Y‐specific markers is common in cell culture,^5^, ^24^ we then investigated the previously reported XX karyotype^12^, ^15^ and examined other methods that might be used for sex determination.

### M14 cells have detectable Y chromosomal material by cytogenetic analysis

Cytogenetic analysis of the M14 cell line was undertaken to better understand the finding that MDA‐MB‐435 has an XX karyotype.^12^, ^15^ Initially, to evaluate X‐ and Y‐chromosomal material dual‐color FISH was performed with a CEP X probe that has homology to the Xp11‐q11 centromeric region (rich in satellite I DNA) and the Yq12 probe that has homology to the heterochromatic Yq12 region (rich in satellite III DNA).

**Figure 3:**
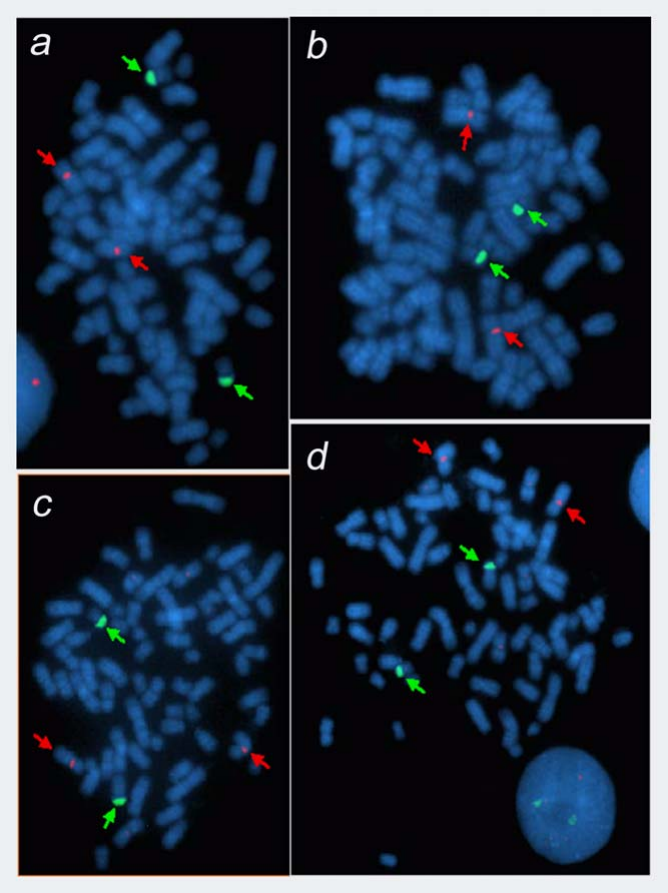
FISH Analysis of M14 using X‐ and Y‐specific probes. Metaphase spreads of M14 cell line, hybridized with the CEP X SpectrumOrange/Yq12 SpectrumGreen FISH probe set (Abbott Molecular). Red arrows indicate Xp11‐q11 and green arrows indicate Yq12 hybridization signals. Images (*a*–*b*) show two similar copies of der(22)t(Y;22), while (*c*–*d*) exhibit two different derivative chromosomes bearing Yq12; namely, the above der(22) and another unknown variant.

**Figure 4:**
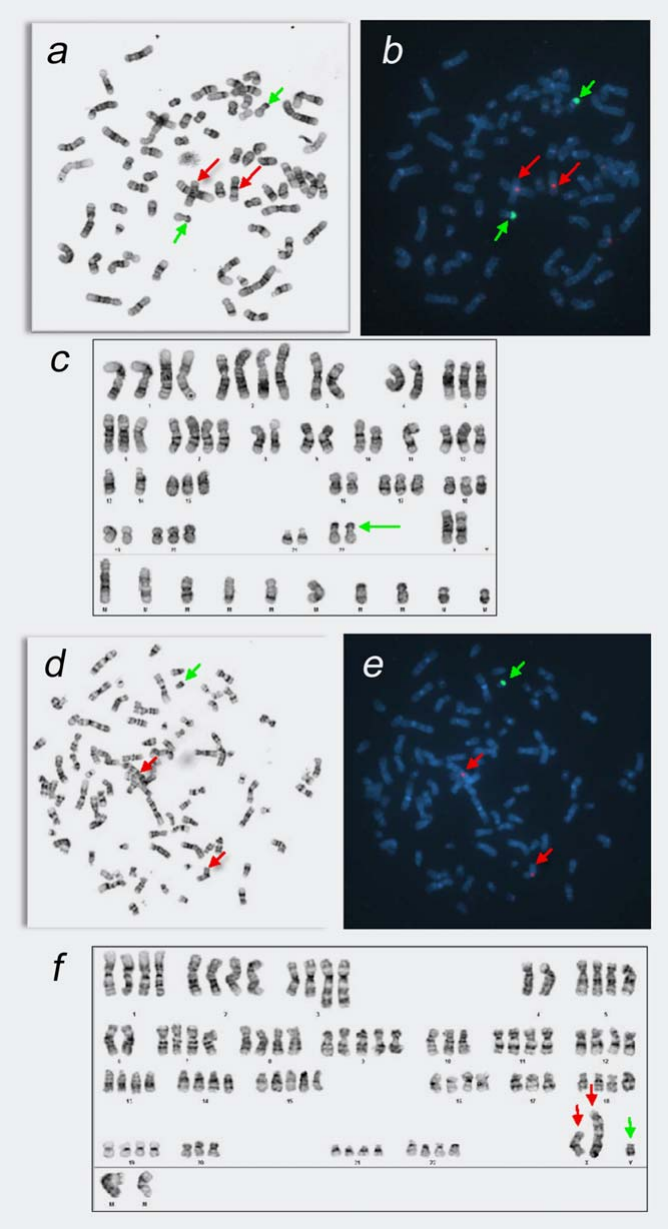
FISH Analysis and Karyotype of M14 and ML14. Metaphase spreads and GTL‐banded karyotype of M14 (*a–c*) and ML14 (*d–f*) cell lines, and the same cells hybridized with the CEP X SpectrumOrange/Yq12 SpectrumGreen FISH probe set (*b*, *e*). Red arrows indicate chromosomes carrying Xp11‐q11 sequences and green arrows indicate chromosomes carrying Yq12 sequences.

Both centromeric X‐ and Yq12‐chromosomal material were detected in M14 (Fig. 3 *a*–3 *d*). Interphase analysis of 100 nuclei showed an average count of 2.4 copies per nucleus for centromere X signals and 1.9 copies for Yq12 signals. Metaphase analyses identified two distinct derivative chromosomes carrying the Yq12 sequences (*e.g*., Figs. 3 *c* and 3 *d*), the shorter derivative being more common than the longer derivative. Sequential G‐banding and FISH analysis revealed the shorter derivative as der(22)t(Y;22)(q12;p11), with the Yq12 material translocated to the short arm of chromosome 22 (Figs. 4 *a* and 4 *c*). The rare, longer derivative, also carrying Yq12 sequences, was not present in the metaphases analyzed by GTL‐banding and FISH.

To determine whether the M14 Y/22 translocation is specific to that cell line, ML14 cells were examined by G‐banding and FISH analysis. Eleven metaphases were karyotyped; representative images are shown in Figures 4 *d*–4 *f*. FISH analysis with the CEP X/Yq12 probe set indicated that each metaphase of the ML14 lymphoblastoid cell line contained a normal X chromosome, a derivative X chromosome with a large unknown fragment added on to its short arm, and a normal Y chromosome. Metaphases were typically tetraploid with numerous rearranged chromosomes.

MDA‐MB‐435S cells were also examined to confirm findings from M14. MDA‐MB‐435S cells displayed two X chromosomes and the Y/22 translocation first seen in M14 (Supporting Information Fig. S4*a*). Counting of interphase nuclei suggested that the Y/22 translocation was present in a minority of cells (Supporting Information Fig. S4*b*).

On the basis of these results, we concluded that both X‐ and Y‐chromosomal material can be detected in M14 and its derivatives. ML14 carries a normal Y chromosome, while M14 displays a Y/22 chromosomal translocation. This translocation does not appear to include amelogenin or the three Y‐SNP loci tested to date (Supporting Information Figs. S1 and S3). We then performed sex‐specific STR analysis to see if other loci were detectable.

### M14 cells have duplicated X chromosomal material but lack Y‐specific STR loci

For the X chromosome, four additional STR loci were examined: DXS‐7132, DXS‐HPRTB, DXS‐7423 and DXS6807. All four X‐specific STR loci were hemi‐ or homozygous in M14 (Table 2) and MDA‐MB‐435 (Supporting Information Table S3). Having already observed that three X‐specific SNP loci were also hemi‐ or homozygous, we concluded that the most likely mechanism for the observed XX karyotype was X‐chromosomal uniparental disomy, with the two X chromosomes having arisen by chromosomal duplication.^34^

**Table 2:** STR analysis of M14 and ML14 using X‐ and Y‐specific STR loci

Cell Line Name	DXS7132	HPRTB	DXS7423	DXS6807	DYS576	DYS389I	DYS448	DYS389II	DYS19	DYS391	DYS481	DYS549	DYS533
(1) ML14	NT	NT	NT	NT	17	13	19	29	14	11	23	13	12
(2) M14	10	13	16	7	ND	ND	ND	ND	ND	ND	ND	ND	ND

**Cell Line Name**	**DYS438**	**DYS437**	**DYS570**	**DYS635**	**DYS390**	**DYS439**	**DYS392**	**DYS643**	**DYS393**	**DYS458**	**DYS385a/b**	**DYS456**	**Y‐GATA‐H4**
(1) ML14	12	15	18	23	24	12	13	11	13	17	11,14	16	12
(2) M14	ND	ND	ND	ND	ND	ND	ND	ND	ND	ND	ND	ND	ND

The first four STR loci in this table are X‐specific and were amplified using a four‐primer pair multiplex PCR designed for X‐STR analysis of cell lines. The remaining STR loci are Y‐specific and were amplified using the Promega PowerPlex^®^ Y23 and PowerPlex^®^ Fusion 6C Systems. Results shown here are from the PowerPlex^®^ Y23 System. Sample sources: (1) ML14 dated January 9,1998, current study (source: JWCI). (2) M14 dated December 2, 1975, current study (source: JWCI). NT = not tested; ND = not detected.

For the Y chromosome, PowerPlex^®^ Y23 and PowerPlex^®^ Fusion 6C kits were used to examine 23 Y‐linked STR loci and additional autosomal loci in ML14 and M14 (Table 2, Supporting Information Table S4). The Y‐STR loci examined are located throughout the Yp and part of the Yq regions (Supporting Information Fig. S1). All 23 Y‐specific STR loci were detectable in ML14 cells but not in M14 cells (Table 2). Loci from the PowerPlex^®^ Fusion 6C System were concordant with data from Identifiler^®^ and PowerPlex^®^ Y23 (Supporting Information Table S4). We concluded that these Y‐specific STR loci are absent from the portion of the Y chromosome that remains in the M14 cell line. Alternatively, STR primer binding site mutations or deletions might be preventing their amplification. It is likely that apart from Yq12, all other Y‐chromosomal material has been lost from M14, either as part of the disease process or during cell line establishment.

## Discussion

Authentication testing of M14 from 1975 (prior to the establishment of MDA‐MB‐435), with comparison to donor serum and lymphoblastoid cell line ML14, shows that M14 is the authentic cell line and MDA‐MB‐435 is a misidentified derivative.

Misidentification of MDA‐MB‐435 is likely to have occurred prior to 1981–1982.^4^ The originators of both cell lines published a paper together that was accepted in May 1979,^35^ which suggests the possibility that each laboratory had access to the other's cell lines at that point in time. It is interesting to note that derivative MDA‐MB‐435S was first deposited at a cell bank in 1982. The deposit records from the originator noted that MDA‐MB‐435S was similar to the original tumor until a spindle‐shaped, faster growing cell emerged (historical deposit information, HTB‐129). This comment is consistent with the first publication to use MDA‐MB‐435S, demonstrating that it grew rapidly with a doubling time of 14–30 hrs.^36^ In retrospect, it seems likely that this faster growing variant represented cross‐contamination and was subsequently distributed by the originator to colleagues and cell line repositories. These colleagues included Dr Janet Price, who received MDA‐MB‐435 from the originator^37^ and later provided it to other laboratories.^3^, ^9^, ^11^, ^38^

Our results are consistent with studies performed previously, using STR profiling to examine authenticity, as well as expression profiling and mutation status to examine tissue origin.^1^, ^2^, ^3^, ^32^ These studies concluded that M14 was the authentic cell line and was derived from melanoma, despite other studies that have demonstrated expression of breast‐specific markers.^9^, ^10^, ^11^, ^38^

Expression of markers that are inconsistent with tissue identity has been explored previously in M14 and MDA‐MB‐435.^2^, ^10^ Expression can vary with cell density, suggesting that growth and assay conditions play an important role in expression of tissue‐specific markers in cell culture.^38^ It should be noted that melanoma can display marked phenotypic diversity, making diagnosis challenging even in a clinical setting.^39^ Similarly, breast carcinoma has been demonstrated to express melanocytic markers, with increased expression in less differentiated tumors.^40^ Lineage infidelity can potentially affect both breast carcinoma and melanoma cells. Phenotype may be helpful in determining tissue origin, but is not sufficient to prove cell line identity. A genotype‐based method of testing is essential for unambiguous identity determination and should be incorporated into policy for authentication of human cell lines, with STR profiling as the consensus for comparison.^22^ The International Journal of Cancer has stringent requirements for authentication testing in place and this investigation demonstrates their importance.^41^

Work on M14 melanoma cells rests on a strong foundation laid down by Dr. Donald Morton and his colleagues in the 1970s. Their preservation of serum and other cell lines from this donor, which were authenticated using methods available at that time, made our subsequent detective work possible. Documentation of cell line provenance, and retention of donor material and early passage samples are essential to prove authenticity. New cell lines should always have donor tissue stored for testing and comparison to later passage samples. Deposit of samples in a core facility or cell line repository will ensure that valuable samples are retained for future use. Deposit of STR profiles into a shared database of cell line STR profiles, such as BioSample,^42^ is also an essential step for future comparison.

Early authentication testing of M14 was performed using HLA typing.^19^ Although HLA typing continues to be used for authentication, it can be difficult to compare early results based on serology^19^ with later results from sequence‐based typing.^43^ STR profiling of early passage samples offers an effective solution, enabling comparison of samples from different laboratories, and its use as a consensus method^22^ has resulted in multiple samples for comparison (Supporting Information Table S1). For example, the STR profiles from other cell lines previously reported to share a common donor origin with MDA‐MB‐435 also corresponded to serum from the melanoma donor, with percent match values of 77–95% (Supporting Information Table S1). These other lines were purportedly derived from papillary thyroid carcinoma and uveal melanoma (Supporting Information Fig. S5, Supporting Information Table S1).

SNP profiling is a complementary approach to STR analysis, although at least one incorrect allele call was detected here, showing the need to optimize the SNP Trace™ System for cell line analysis. Cytogenetic analysis can also be highly informative. For this study, it was the only method to detect the Y‐chromosomal material in M14 cells.

Consideration of sex in the design and interpretation of preclinical studies is important and has been highlighted by NIH and others.^44^, ^45^ Clinical outcomes in melanoma can vary with the sex of the patient, suggesting that this variable is relevant for melanoma research.^46^ Oncogenes and tumor suppressors are present on X and Y chromosomes, and have been proposed as biological mechanisms to explain the sex disparities in melanoma. For example, the TSPY oncogene on the Y chromosome is known to be dysregulated in some melanoma cell lines.^46^

Donor sex is therefore relevant, but can be difficult to determine in cell lines. Loss of Y‐chromosomal material is frequently seen in cell culture. A recent study of 1,843 human cell lines demonstrated that 331 were annotated as male but called as female through absence of AMELY on STR analysis.^24^ Our analysis of donor serum and ML14 demonstrated that M14 and MDA‐MB‐435 come from a male donor. Cytogenetic analysis showed that M14 and MDA‐MB‐435S cells carry a derivative chromosome 22 from a translocation with Yq12. However, despite further STR and SNP analysis, we were unable to detect Y‐specific loci in either cell line.

The absence of Y‐specific loci suggests that the majority of the Y chromosome has been lost from M14 and MDA‐MB‐435 cells. Y chromosomal rearrangements may have occurred during cell line establishment or ongoing culture. Variable Y‐chromosome numbers have been observed while examining A549 cells from different sources using cytogenetic analysis.^47^ Y translocations have been documented in cell lines, for example, in prostate cell line DU 145.^48^ Y/22 translocations can also be associated with cancer, usually in association with a variant Philadelphia chromosome.^49^ Loss of Y chromosomal material with retention of heterochromatin has been seen in melanoma previously.^50^

X‐chromosomal uniparental disomy is the most likely mechanism for the XX karyotype previously reported for M14 and MDA‐MB‐435.^12^, ^15^ Duplication of X‐chromosomal material has been demonstrated previously in male cell lines established from prostate cancer.^28^ Cytogenetic analysis showed that ML14 cells carry X chromosomal rearrangements. This is an unusual finding for lymphoblastoid cell lines, where tetraploidy is common, but rarely produces genetic imbalances.^51^ Although lymphoblastoid cell lines from normal patients typically have stable karyotypes, chromosomal abnormalities have been documented in association with immortalization.^52^

We conclude that the M14 cell line is authentic, established from a male donor with melanoma. Currently available stocks of MDA‐MB‐435 are misidentified and were derived from M14. Unless authentic stocks of MDA‐MB‐435 can be identified, investigators should be aware that MDA‐MB‐435 is not a female cell line and is not a suitable model for breast carcinoma. Cell lines are living materials and can be complex, changeable, and difficult to understand. Clear provenance and careful characterization of widely used cell lines, such as M14 and MDA‐MB‐435, are essential if we are to understand the tumors from which they were established.

## Supporting information

Supplementary Material

Supplementary Tables

## References

[1] Ross DT , Scherf U , Eisen MB , et al. Systematic variation in gene expression patterns in human cancer cell lines. Nat Genet 2000;24:227–35. 10700174 10.1038/73432

[2] Ellison G , Klinowska T , Westwood RF , et al. Further evidence to support the melanocytic origin of MDA‐MB‐435. Mol Pathol. 2002;55:294–9. 12354931 10.1136/mp.55.5.294 PMC1187258

[3] Rae JM , Ramus SJ , Waltham M , et al. Common origins of MDA‐MB‐435 cells from various sources with those shown to have melanoma properties. Clin Exp Metastasis 2004;21:543–52. 15679052 10.1007/s10585-004-3759-1

[4] Rae JM , Creighton CJ , Meck JM , et al. MDA‐MB‐435 cells are derived from M14 melanoma cells–a loss for breast cancer, but a boon for melanoma research. Breast Cancer Res Treat 2007;104:13–9. 17004106 10.1007/s10549-006-9392-8

[5] Lorenzi PL , Reinhold WC , Varma S , et al. DNA fingerprinting of the NCI‐60 cell line panel. Mol Cancer Ther 2009;8:713–24. 19372543 10.1158/1535-7163.MCT-08-0921 PMC4020356

[6] Christgen M , Lehmann U. MDA‐MB‐435: the questionable use of a melanoma cell line as a model for human breast cancer is ongoing. Cancer Biol Ther 2007;6:1355–7. 17786032 10.4161/cbt.6.9.4624

[7] Lacroix M. Persistent use of “false” cell lines. Int J Cancer 2008;122:1–4. 17960586 10.1002/ijc.23233

[8] Prasad VV , Gopalan RO. Continued use of MDA‐MB‐435, a melanoma cell line, as a model for human breast cancer, even in year, 2014. Npj Breast Cancer 2015;1:1–2. 10.1038/npjbcancer.2015.2 PMC5515196 28721362

[9] Sellappan S , Grijalva R , Zhou X , et al. Lineage infidelity of MDA‐MB‐435 cells: expression of melanocyte proteins in a breast cancer cell line. Cancer Res 2004;64:3479–85. 15150101 10.1158/0008-5472.CAN-3299-2

[10] Zhang Q , Fan H , Shen J , et al. Human breast cancer cell lines co‐express neuronal, epithelial, and melanocytic differentiation markers in vitro and in vivo. PLoS One 2010;5:e9712. 10.1371/journal.pone.0009712 PMC2838789 20300523

[11] Montel V , Suzuki M , Galloy C , et al. Expression of melanocyte‐related genes in human breast cancer and its implications. Differentiation 2009;78:283–91. 19699574 10.1016/j.diff.2009.07.007

[12] Chambers AF. MDA‐MB‐435 and M14 cell lines: identical but not M14 melanoma? Cancer Res 2009;69:5292–3. 19549886 10.1158/0008-5472.CAN-09-1528

[13] Wong JH , Aguero B , Gupta RK , et al. Recovery of a cell surface fetal antigen from circulating immune complexes of melanoma patients. Cancer Immunol Immunother 1988;27:142–6. 3046746 10.1007/BF00200019 PMC11038578

[14] Cailleau R , Olive M , Cruciger QV. Long‐term human breast carcinoma cell lines of metastatic origin: preliminary characterization. In Vitro 1978;14:911–5. 730202 10.1007/BF02616120

[15] Hollestelle A , Schutte M. Comment Re: MDA‐MB‐435 and M14 cell lines: identical but not M14 Melanoma? Cancer Res 2009;69:7893 19723654 10.1158/0008-5472.CAN-09-2396

[16] Liang‐Chu MM , Yu M , Haverty PM , et al. Human biosample authentication using the high‐throughput, cost‐effective SNPtrace(TM) system. PLoS One 2015;10:e0116218. 10.1371/journal.pone.0116218 PMC4340925 25714623

[17] Boyd MR , Paull KD. Some practical considerations and applications of the national cancer institute in vitro anticancer drug discovery screen. Drug Dev Res 1995;34:91–109.

[18] Chee DO , Boddie AW , Roth JA , et al. Production of melanoma‐associated antigen(s) by a defined malignant melanoma cell strain grown in chemically defined medium. Cancer Res 1976;36:1503–9. 1260767

[19] Saxton RE , Irie RF , Ferrone S , et al. Establishment of paired tumor cells and autologous virus‐transformed cell lines to define humoral immune responses in melanoma and sarcoma patients. Int J Cancer 1978;21:299–306. 204583 10.1002/ijc.2910210308

[20] Orchard G. Evaluation of melanocytic neoplasms: application of a pan‐melanoma antibody cocktail. Br J Biomed Sci 2002;59:196–202. 12572952 10.1080/09674845.2002.11783659

[21] Yamamoto F , Clausen H , White T , et al. Molecular genetic basis of the histo‐blood group ABO system. Nature 1990;345:229–33. 2333095 10.1038/345229a0

[22] ANSI/ATCC ASN‐0002–2011. Authentication of human cell lines: Standardization of STR profiling ANSI eStandards Store, 2012 Available at: http://webstore.ansi.org/RecordDetail.aspx?sku=ANSI%2fATCC+ASN-0002-2011.

[23] Capes‐Davis A , Reid YA , Kline MC , et al. Match criteria for human cell line authentication: Where do we draw the line? Int J Cancer 2013;132:2510–9. 23136038 10.1002/ijc.27931

[24] Yu M , Selvaraj SK , Liang‐Chu MM , et al. A resource for cell line authentication, annotation and quality control. Nature 2015;520:307–11. 25877200 10.1038/nature14397

[25] Promega Corporation . PowerPlex(R) Y23 System Technical Manual, 2015 Available at: www.promega.com/~/media/files/resources/protocols/technical%20manuals/101/powerplex%20y23%20system%20protocol.pdf. Accessed 29 September 2017.

[26] Promega Corporation . PowerPlex(R) Fusion 6C System Technical Manual www.promega.com/~/media/files/resources/protocols/technical%20manuals/101/powerplex%20fusion%206c%20system%20protocol.pdf, 2015. Accessed 29 September 2017.

[27] Bunn PA, Jr , Helfrich B , Soriano AF , et al. Expression of Her‐2/neu in human lung cancer cell lines by immunohistochemistry and fluorescence in situ hybridization and its relationship to in vitro cytotoxicity by trastuzumab and chemotherapeutic agents. Clin Cancer Res 2001;7:3239–50. 11595720

[28] van Bokhoven A , Caires A , Maria MD , et al. Spectral karyotype (SKY) analysis of human prostate carcinoma cell lines. Prostate 2003;57:226–44. 14518030 10.1002/pros.10291

[29] Brinkley BR , Beall PT , Wible LJ , et al. Variations in cell form and cytoskeleton in human breast carcinoma cells in vitro. Cancer Res 1980;40:3118–29. 7000337

[30] Hoon DS. Donald Lee Morton: in memoriam (1934–2014). Cancer Res 2014;74:4009–10. 25229088 10.1158/0008-5472.can-14-1615

[31] Fang M , Hutchinson L , Deng A , et al. Common BRAF(V600E)‐directed pathway mediates widespread epigenetic silencing in colorectal cancer and melanoma. Proc Natl Acad Sci USA 2016;113:1250 26787892 10.1073/pnas.1525619113 PMC4747705

[32] McDermott U , Sharma SV , Dowell L , et al. Identification of genotype‐correlated sensitivity to selective kinase inhibitors by using high‐throughput tumor cell line profiling. Proc Natl Acad Sci USA 2007;104:19936–41. 18077425 10.1073/pnas.0707498104 PMC2148401

[33] Lee JE , Hong EJ , Kim JH , et al. Instability at short tandem repeats in lymphoblastoid cell lines. Osong Public Health Res Perspect 2013;4:194–6. 24159555 10.1016/j.phrp.2013.06.003 PMC3767104

[34] Spence JE , Perciaccante RG , Greig GM , et al. Uniparental disomy as a mechanism for human genetic disease. Am J Hum Genet 1988;42:217–26. 2893543 PMC1715272

[35] Higuchi M , Robinson DS , Cailleau R , et al. A serologic study of cultured breast cancer cell lines: lack of antibody response to tumour specific membrane antigens in patients. Clin Exp Immunol 1980;39:90–6. 7389200 PMC1537957

[36] McCormack SA , Bearden D , Dennison DK , et al. Methodological aspects of analysing human breast cancer cell lines by NMR spectroscopy. Physiol Chem Phys Med NMR 1984;16:359–79. 6531402

[37] Price JE , Polyzos A , Zhang RD , et al. Tumorigenicity and metastasis of human breast carcinoma cell lines in nude mice. Cancer Res 1990;50:717–21. 2297709

[38] Nerlich AG , Bachmeier BE. Density‐dependent lineage instability of MDA‐MB‐435 breast cancer cells. Oncol Lett 2013;5:1370 23599796 10.3892/ol.2013.1157 PMC3629269

[39] Bacchi CE , Wludarski SC , Ambaye AB , et al. Metastatic melanoma presenting as an isolated breast tumor: a study of 20 cases with emphasis on several primary mimickers. Arch Pathol Lab Med 2013;137:41–9. 23276173 10.5858/arpa.2011-0552-OA

[40] Bachmeier BE , Nerlich AG , Mirisola V , et al. Lineage infidelity and expression of melanocytic markers in human breast cancer. Int J Oncol 2008;33:1011–5. 18949364

[41] Fusenig NE , Capes‐Davis A , Bianchini F , et al. The need for a worldwide consensus for cell line authentication: Experience implementing a mandatory requirement at the International Journal of Cancer. PLoS Biol 2017;15:e2001438. 10.1371/journal.pbio.2001438 PMC5393552 28414712

[42] Barrett T , Clark K , Gevorgyan R , et al. BioProject and BioSample databases at NCBI: facilitating capture and organization of metadata. Nucleic Acids Res 2012;40:D57–63. 22139929 10.1093/nar/gkr1163 PMC3245069

[43] Adams S , Robbins FM , Chen D , et al. HLA class I and II genotype of the NCI‐60 cell lines. J Transl Med 2005;3:11 15748285 10.1186/1479-5876-3-11 PMC555742

[44] Clayton JA , Collins FS. Policy: NIH to balance sex in cell and animal studies. Nature 2014;509:282–3. 24834516 10.1038/509282a PMC5101948

[45] Shah K , McCormack CE , Bradbury NA. Do you know the sex of your cells?. Am J Physiol, Cell Physiol 2014;306:C3–18. 24196532 10.1152/ajpcell.00281.2013 PMC3919971

[46] Nosrati A , Wei ML. Sex disparities in melanoma outcomes: the role of biology. Arch Biochem Biophys 2014;563:42–50. 25057772 10.1016/j.abb.2014.06.018

[47] Honma M , Hayashi M , Ohno T , et al. Heterogeneity of the Y chromosome following long‐term culture of the human lung cancer cell line A549. In Vitro Cell Dev Biol Anim 1996;32:262–4. 8792154 10.1007/BF02723057

[48] Pan Y , Kytola S , Farnebo F , et al. Characterization of chromosomal abnormalities in prostate cancer cell lines by spectral karyotyping. Cytogenet Genome Res 1999;87:225–32. 10.1159/000015432 10702678

[49] Gallego MS , Baialardo EM , Gutierrez M , et al. New variant Ph translocation in chronic myeloid leukemia: t(Y;22)(p11;q11). Cancer Genet Cytogenet 1996;87:75–8. 8646747 10.1016/0165-4608(95)00231-6

[50] Doneda L , Larizza L. Loss of Y chromosome with retention of Y heterochromatin in a marker chromosome from a human melanoma. Int J Cancer 1991;47:154–7. 1985872 10.1002/ijc.2910470127

[51] MacLeod RA , Drexler HG. Cytogenetic characterization of tumor cell lines. Methods Mol Med 2004;88:57–76. 14634218 10.1385/1-59259-406-9:57

[52] Sugimoto M , Tahara H , Ide T , et al. Steps involved in immortalization and tumorigenesis in human B‐lymphoblastoid cell lines transformed by Epstein‐Barr virus. Cancer Res 2004;64:3361–4. 15150084 10.1158/0008-5472.CAN-04-0079

