# Supplementary material for: Authentication of M14 melanoma cell line proves misidentification of MDA‐MB‐435 breast cancer cell line
Source: Int J Cancer. 2017 Oct 10;142(3):561–72. doi: 10.1002/ijc.31067 (PMC5762610; doi:10.1002/ijc.31067)
Supplement: Supplementary file 1 — Supplementary Material [file IJC-142-561-s001.docx]

# Supplementary Table and Figure Legends

## Supplementary Table S1. STR analysis of Synonymous Cell Lines

Cell lines are listed that share a common donor origin with M14 (synonymous cell lines), based on STR profile comparison. Data include STR profiles generated for the current study using the ABI Identifiler^®^ kit, and profiles obtained from public sources, including journal articles and online databases of human cell line STR profiles. All sources are listed in the table. The sample selected as a reference for comparison is highlighted in green; alleles that differ from the reference sample are highlighted in orange.

## Supplementary Table S2. SNP analysis using SNP Trace™ Panel.

SNP loci are listed for M14, ML14, and MDA-MB-435 cell lines using the SNP Trace™ Panel. Data include SNP profiles generated for the current study and a SNP profile from a previously published study. All sources are listed in the table. The sample selected as a reference for comparison is highlighted in green; alleles that differ from the reference sample are highlighted in orange.

## Supplementary Table S3. X-STR analysis (DXS6807, DXS7132, DXS7423, HPRTB)

X-specific STR loci are listed for M14 and MDA-MB-435 cell lines using a novel DXS multiplex PCR. STR data for M14 have previously been reported (Table 2); the Supplementary table contains additional control samples.

## Supplementary Table S4. STR analysis using PowerPlex® Fusion 6C System

STR profiles are listed for M14 and ML14 cell lines using the PowerPlex^®^ Fusion 6C System. The STR loci included in the PowerPlex^®^ Fusion 6C System have previously been reported (Table 2) and are supplied here to demonstrate concordance.

## Supplementary Figure S1. Locations of Y-STR and Y-SNP loci in the current study


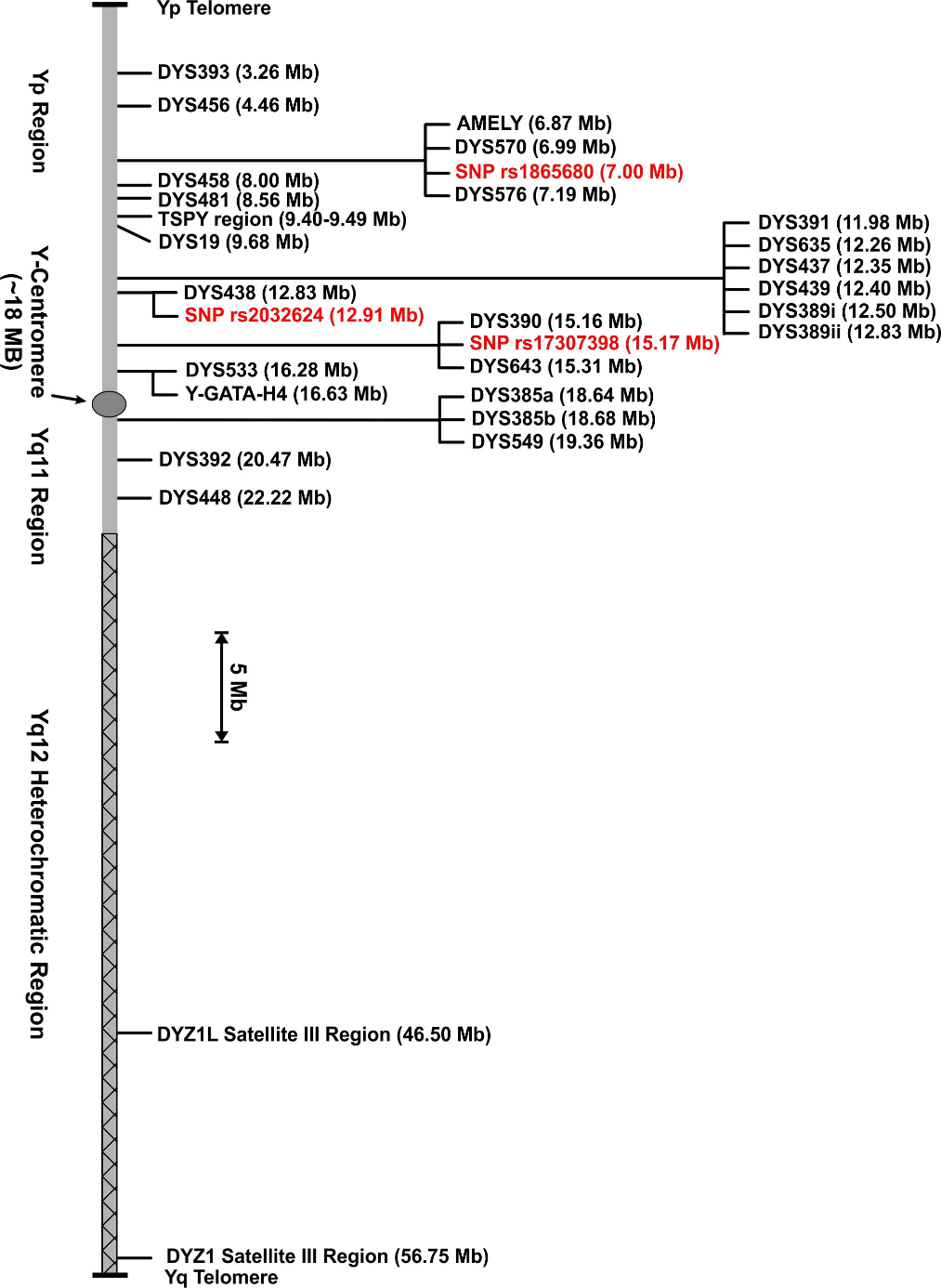


A map of the *Homo sapiens* Y chromosome, demonstrating the locations of the Y-specific loci used in the current study. We compiled this map of the human Y chromosome based on version GRCh38.p7 Primary Assembly DNA Sequence of the human genome and Y-STR information from Butler et al. (1). The three Y-linked SNPs detected by the SNP Trace™ System (rs1865680, rs2032624, rs17307398) are indicated in red. The approximate midpoints of the 23 STR loci detected by Promega's PowerPlex® Y23 System, and other commonly used Y-specific loci (such as amelogenin) are indicated in black. Mb = megabasepairs.

## Supplementary Figure S2. Sequencing of X-SNP loci


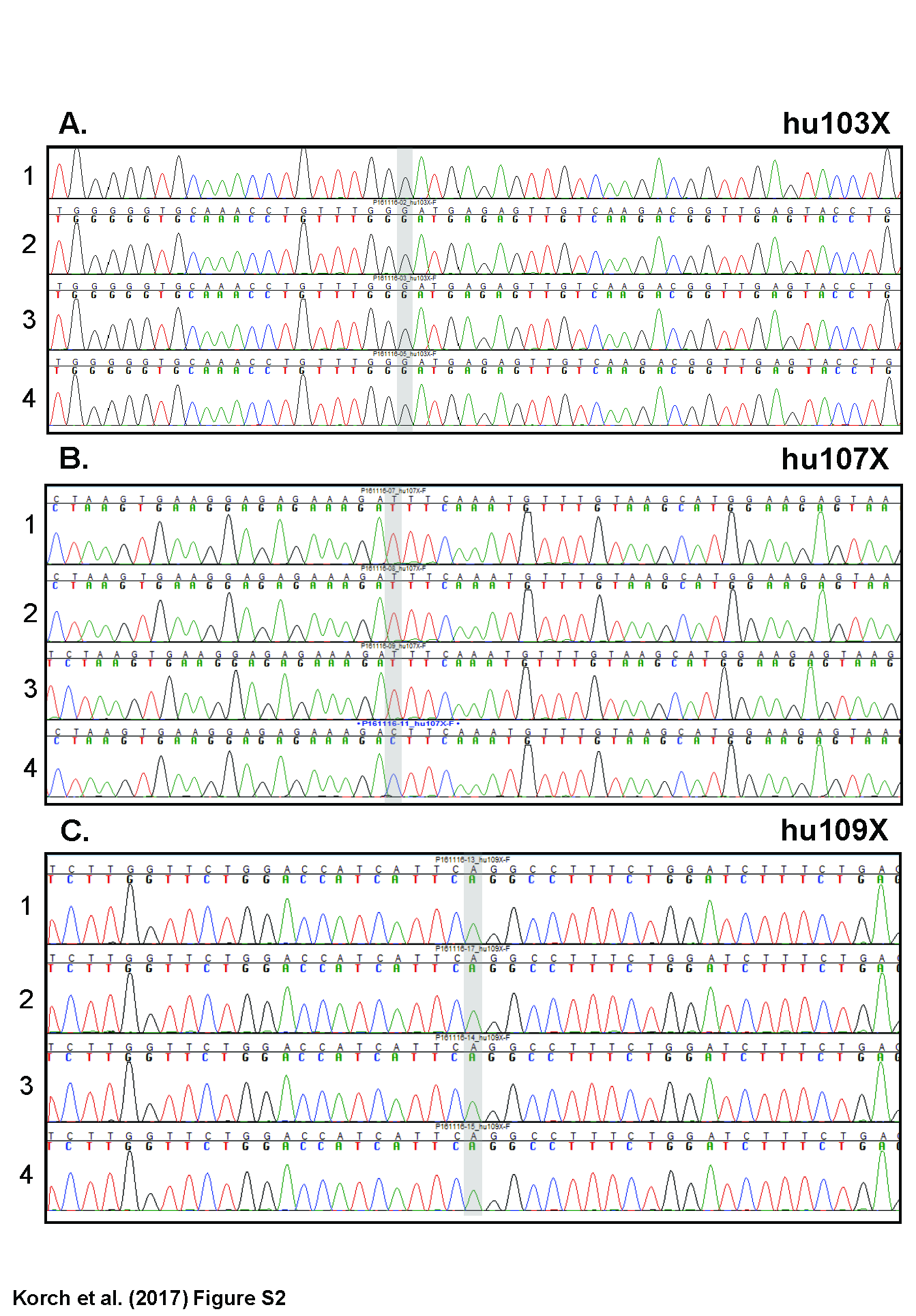


Sequence data corresponding to X-specific SNP loci in the Fluidigm SNP Trace™ Panel. SNP loci seen here are A) hu103X (rs525869); B) hu107X (rs2040962); and C) hu109X (rs530501). Grey shading indicates the precise location of the SNP locus in each case. Samples correspond to 1) M14 cells; 2) ML14 lymphoblastoid cells; 3) MDA-MB-435S cells; and 4) EM42 (HeLa), which was used as a female control. It should be noted that hu103X, which was called as heterozygous when analyzed using the SNP Trace™ Panel, is homo- or hemizygous when sequenced in all samples.

## Supplementary Figure S3. PCR amplification of Y-SNP loci and autosomal controls


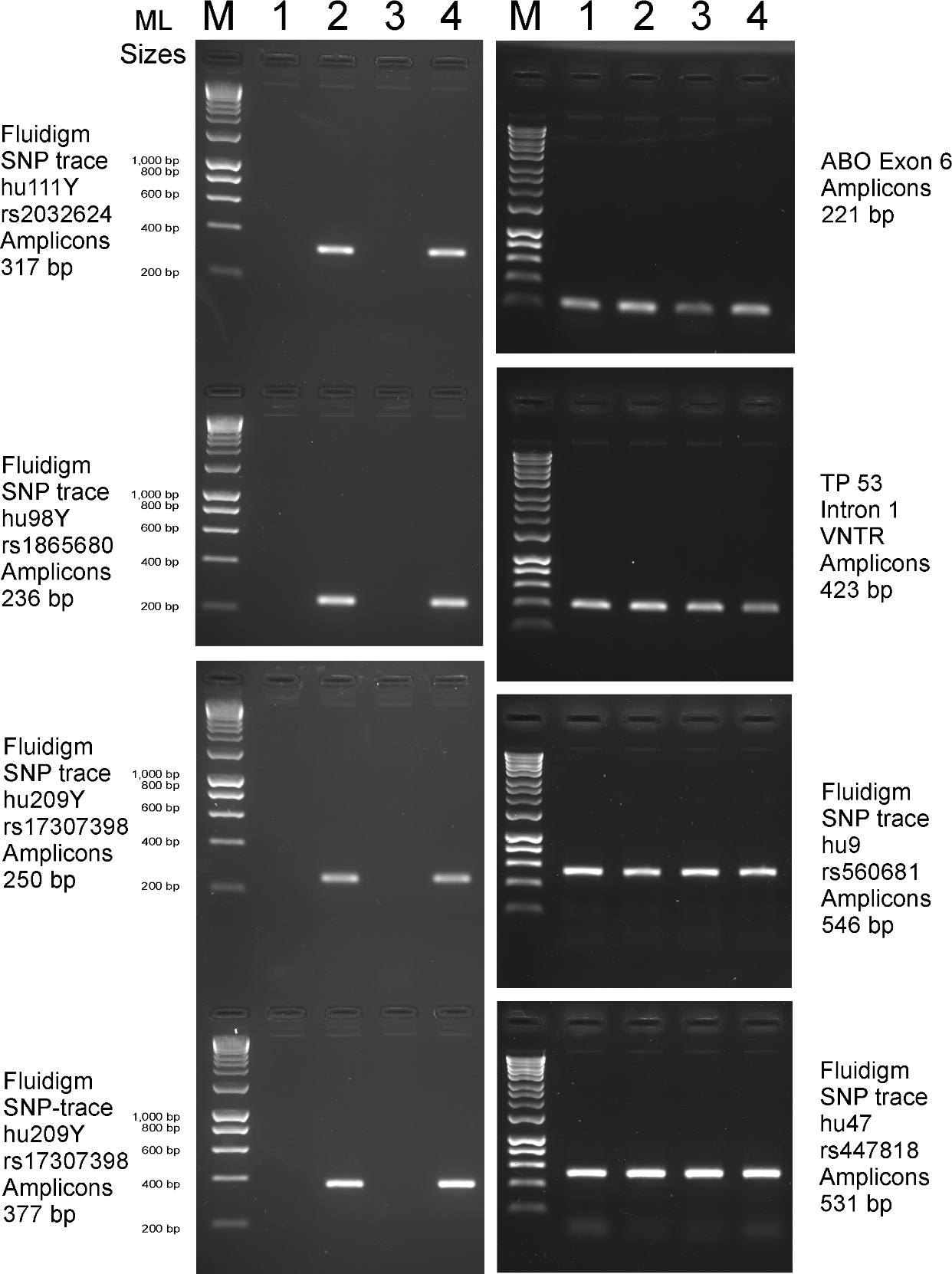


PCR amplification of Y-SNP loci and autosomal controls, visualized using agarose gel electrophoresis. Sample lanes are as follows: M = Size marker; 1 = M14 (JWCI, passage 16); 2 = ML14 (JWCI, passage unknown); 3 = MDA-MB-435S (ATCC, passage 345); 4 = Promega mixed male DNA sample (catalog number G1471). Regions amplified by PCR included six SNP loci tested by the SNP Trace™ Panel; exon 6 of the ABO gene, containing the blood type O deletion (mRNA allele 258G at position 133,257,523 of chromosome 9); and the VNTR region of intron 1 of the TP53 gene, used as a DNA quality control. Amplification was performed as described below in the Supplementary Methods. The DNA sequence of each PCR product confirmed the SNP Trace™ Panel result.

## Supplementary Figure S4. FISH Analysis of MDA-MB-435S.


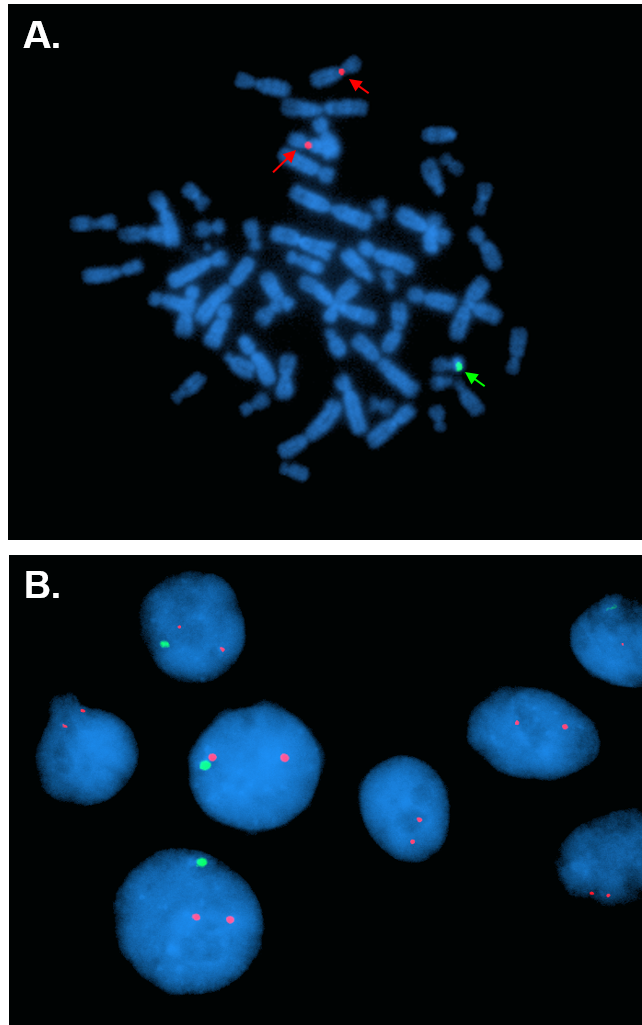


Analysis of MDA-MB-435S using the CEP X SpectrumOrange/Yq12 SpectrumGreen FISH probe set (Abbott Molecular). A) Representative metaphase showing two copies of chromosome X (red arrows) and one Y-chromosomal rearrangement (der(22)(22;Y)(p12;q12), green arrow). B) Representative interphase cells showing two copies of chromosome X (red signals), with either zero (76%), one (17%), or two (7%) copies of Yq12 chromosomal material (green signals, n=100).

## Supplementary Figure S5. M14 Family of Synonymous Cell Lines





Authentication testing by STR genotyping (Supplementary Table S1) shows that multiple cell lines share a common donor origin with the M14 cell line. These can be derived into three groups based on their purported origin. Cell lines were reported to originate from metastatic breast cancer, multiple papillary thyroid tumors, and two uveal melanomas. The NPA'87 and BHP series of papillary thyroid cell lines probably arose from M14, because the originating laboratory had used the M14 cell line (see source reference, Supplementary Table S1). The uveal melanoma cell lines probably arose from M14, considering that the originating laboratories studied melanoma.

# Supplementary Methods

## Immunostaining using the Benchmark XT® automated stainer

Immunostaining was performed using the Benchmark XT® automated stainer. Equipment and reagents were supplied by Ventana Medical Systems (Tucson, AZ USA, catalogue numbers listed below in brackets). Steps included:

1. Application of Cell Conditioning Solution (CCS1, catalogue number 950-124) for 64 minutes.

2. Application of ready-to-use Melanoma Triple Cocktail Primary Antibody (catalogue number 790-4677) and incubation at 37^o^C for 16 minutes.

3. Application of ultraView Universal DAB Detection Kit (catalogue number 760-500) for antibody detection, in accordance with the manufacturer’s instructions.

4. Counterstaining with Hematoxylin for 4 minutes and post-counterstaining with Bluing Reagent (catalogue number 760-2037) for 4 minutes.

5. Washing of slides with mild soapy water, followed by dehydration in a series of 70% to 100% ethanol and xylene baths.

## Extraction of DNA from donor serum

This procedure was adapted from a previous publication that described extraction of circulating DNA from human serum (2). Increased amounts of circulating DNA have been observed in cancer patients (3).

Serum (30-40 µL) was heated at 95°C for 5 minutes and cooled. This resulted in coagulation of the serum, with formation of a clump of protein. Water (20-30 µL) was added and the clump was macerated with a pipette tip, vigorously vortexed, and centrifuged at 10,000 x *g* for 5 minutes. The supernatant was removed and 1-10 µL used for PCR amplification. The coagulation step and elution from the clump appears to provide a "cleaner" solution that is less inhibitory. The amount used for amplification is also important; more than 4 µL appears to inhibit the PCR.

## Step by step workflow for comparison of STR and SNP profiles

This workflow was used to compare STR profiles in Supplementary Table S1 and SNP profiles in Supplementary Table S2. When comparing STR or SNP profiles:

1. A Test sample is selected for comparison.

2. A Reference sample is selected for comparison. Reference samples are highlighted in green.

3. The number of loci in common for both profiles ("Common Loci") are counted and recorded. Alleles are counted only at these “Common Loci”.

4. The total number of distinct alleles in the Test sample ("Total Alleles Test") are counted and recorded.

5. The total number of distinct alleles in the Reference sample ("Total Alleles Reference") are counted and recorded.

6. The number of shared alleles in Test and Reference sample ("Shared Alleles") are counted and recorded.

7. “Percent match” is calculated using the following algorithm: Percent match = (Shared Alleles x 2)/(Total Alleles Test + Total Alleles Reference). The result is expressed as a percentage.

8. Results are interpreted using match criteria set out in Capes-Davis et al. (4) and Yu et al. (5) and entered in the column labelled “Comment”.

9. Results in the table are sorted based on percent match, from high to low values.

For more information, see the ICLAC worksheet on Match Criteria for Human Cell Line Authentication: <http://iclac.org/resources/match-criteria-worksheet/>

## X-STR analysis (DXS6807, DXS7132, DXS7423, HPRTB)

This procedure was used to analyze the X-specific STR loci in Table 2 and Supplementary Table S3.

### Preparation of high molecular weight DNA

The M14 DNA was prepared using the Zymo Research Genomic DNA mini-extraction kit (D3024). DNA from the control samples for analysis of X-linked STR loci was prepared as follows. A cell culture suspension containing 3-5 x 10^6^ diploid cells was centrifuged at 2,000 x *g* for 4 minutes. The supernatant was removed with a disposable pipette and discarded. The remaining pellet was carefully resuspended in 5 mL PBS. After the washing step, the pellet was resolved in 200 µL PBS by vortexing, taking care to resuspend even tiny clumps of cells prior to lysis. For isolation of other genomic DNA, the High Pure PCR Template Preparation Kit was used (Roche, Mannheim Germany; catalogue number 11796828001). In our hands, 200 µL lysis buffer was added to the sample solution and mixed by pipetting. Immediately after lysis, 40 µL proteinase K was added and mixed well using a vortex, followed by incubating at 72°C for 30 minutes. After adding 100 µL of isopropanol, the sample was well mixed and applied to a filter tube. Centrifugation was carried out for 1 minute at 8,000 *g* and the flow-through discarded. After addition of 500 µL of inhibitor removal buffer, the filter tube was re-centrifuged for 1 min at 8,000 *g*. Upon discarding the flow-through, 500 µL of wash buffer was added and centrifuged for 1 minute at 8,000 *g*. This step was repeated. After supplying a new tube and 200 µL of elution buffer preheated to 72°C, the filter tube was finally centrifuged for 1 minute at 8,000 *g*. For maximum yield, the elution step was repeated using 100 µL elution buffer. The purified genomic DNA concentration was approximately 10 ng/µL per sample, depending on the ploidy status of the cell line used. The genomic DNA was stored at 4°C.

### Hot start quadruplex fluorescence DXS DNA typing

The X-chromosome-specific DXS quadruplex PCR described here contains four different STR primer pairs. The following procedure and parameters were optimized for 0.2 mL reaction tubes in an i-Cycler (Bio-Rad). It is fundamentally important to integrate the appropriate positive and negative controls (e. g., HeLa DNA template and H_2_O as no template control, respectively). A pre-master mix calculated for 25 µL per reaction of each sample was prepared with enough for one additional reaction. For a single reaction, the components are as follows:

Primer pairs were adjusted to 100 µM and a working DXS primer solution was prepared, to give a 2.5 pmol solution of each primer pair (sequence listed below) in 400 µL total volume. For starting the PCR, a master mix was prepared containing (per sample) 1 µl of working DXS primer solution, 2.5 µl 10 x Hot start PCR buffer (any supplier), 1 µl dNTP (5 µM), 0.2 µl (1 unit) Hot start Taq polymerase (any supplier), and 19.8 µl distilled water. Genomic DNA (1 µl adjusted to 10 ng/µl) was added after, and independent from, the master mix solution.

The PCR program was as follows:

Cycle 1: 95°C for 3 min - 1 repeat.

Cycle 2: 94°C for 30 sec, 57°C for 30 sec, 72°C for 45 sec - 30 repeats.

Cycle 3: 60°C for 15 min - 1 repeat.

### X-STR primer sequences

All forward primers were fluorescently labelled using WellRED D4 dye (Beckmann Coulter, Krefeld Germany; catalogue number 608145).

#### DXS6807 (6)

PrimerF: 5' - GAGCAATGATCTCATTTGCA - 3'

PrimerR: 5' - AAGTAAACATGTATAGGAAAAAGCT - 3'

Polymorphism consists of seven alleles with amplicons in the size range 251-275 bp.

#### HPRTB (7-8)

PrimerF: 5' - TCTCTATTTCCATCTCTGTCTCC - 3'

PrimerR: 5' - TCACCCCTGTCTATGGTCTCG - 3'

Polymorphism consists of nine alleles with amplicons in the size range 144-176 bp.

#### DXS7132 (7,9)

PrimerF: 5' - GAGCCCATTTTAATAAATCC - 3'

PrimerR: 5' - GCCAAACTCTATTAGTCAAC - 3'

Polymorphism consists of seven alleles with amplicons in the size range 131-155 bp.

#### DXS7423 (7,9-10)

PrimerF: 5' - GTCTTCCTGTCATCTCCCAAC - 3'

PrimerR: 5' - AGCTTAGCGCCTGGCACATA - 3'

Polymorphism consists of seven alleles with amplicons in the size range 200-228 bp.

### DXS PCR fragment detection and analysis

In a microtiter plate, aliquots of 1 µl of the amplification products were combined with 0.25 µl of an internal size standard (Size standard kit 400, Beckman-Coulter) in a total volume of 30 µl of sample loading solution. The samples were automatically loaded and analyzed using the capillary electrophoresis system CEQ 8000 (Beckman-Coulter) using fragment analysis parameters, established over a 6 month period of DNA typing of X chromosomes and with bio-computational help at <http://www.chrx-str.org/>. Fragment analysis software of CEQ 8000 enabled the precise size determination of detected alleles resulting in a genotype summary list.

## Sequencing of X-SNP loci

This procedure was used to explore results from the Fluidigm SNP Trace™ Panel. One of the X-linked SNPs in the panel, hu103X (rs525869), was called as heterozygous A/G by the analysis program. This was a discordant result because other data from serum and lymphoblastoid cells demonstrated that samples come from a male donor. Three X-linked SNPs included in the Fluidigm assay were amplified and sequenced to independently assess allele calls: hu103X (rs525869), hu107X (rs2040962), and hu109X (rs530501). X-SNP primer sequences were designed to amplify each region (see below). ML14, M14, MDA-MB-435S and control female DNA from EM42 (a misidentified cell line derived from HeLa) DNA was amplified and forward primers used for sequencing.

### X-SNP primer sequences

#### SNP47 hu103X (rs525869)

hu103X_rs525869-F1 5’ - AGAGGGCTTTGCTTGTTTTATTGTAC - 3’

hu103X_rs525869-R1 5’ - AGCCTTATAAAAATCTTGAAAAGAAGGA - 3’

#### SNP59 hu107X (rs2040962)

#### hu107X_rs2040962-F1 5’ - CAAAGGAGCAAGAGGGATTTTATG - 3’

hu107X_rs2040962-R1 5’ - GGCTCTGATTTTCACTCAATCTAAAG - 3’

#### SNP71 hu109X (rs530501)

hu109X_rs530501-F1 5’ - TGGATGTGTGGGCAACCTGTAT - 3’

hu109X_rs530501-R1 5’ - GAAAGGCCGTGCAGTTACATCA - 3’

## PCR amplification of Y-SNP loci and autosomal controls

This procedure was used to confirm results from the Fluidigm SNP Trace™ Panel, where Y-SNP data were discordant with a previous study (11). Three Y-linked SNPs included in the Fluidigm assay were amplified: hu98Y (rs1865680), hu111Y (rs2032624), and hu209Y (rs17307398). Autosomal loci were amplified as positive controls for DNA quality and success of the PCR. Autosomal loci included the O allele in exon 6 of the ABO blood type locus, containing the allele encoding ABO blood type O; the VNTR (variable number of tandem repeats) alleles in intron 1 of the TP53 gene; and two regions of chromosomes 1 and 6 that encompass autosomal SNP loci in the Fluidigm SNP Trace™ assay, namely hu9 (rs560681) and hu47 (rs447818). The PCR results are shown in Supplementary Figure S2 above.

### Touchdown PCR amplification conditions

Reaction mixtures were prepared with a total volume of 50 µL and consisted of DNA polymerase (ThermoFisher GeneAmp High Fidelity PCR System, catalogue 4328217, 2 units per 50 µL reaction); dNTPs at final concentrations of 200 µM each; primers at final concentrations of 200 nM each; and genomic DNA (10-20 ng per 50 µL reaction). Tubes were placed into an ABI 9700 thermal cycler once the heat block reached approximately 90°C.

The PCR program used for Y-SNP loci was as follows:

Cycle 1: 96^o^C for 3 min - 1 repeat.

Cycle 2: 96^o^C for 30 sec, 65^o^C for 1 min (decreasing from 65^o^C to 55^o^C by 0.5^o^C with each repeat), 68^o^C for 2 min - 20 repeats.

Cycle 3: 96^o^C for 30 sec, 55^o^C for 1 min, 68^o^C for 2 min - 15 repeats.

Cycle 4: 68^o^C for 15 min, hold at 10^o^C - 1 repeat.

The PCR program used for autosomal loci was as follows:

Cycle 1: 96^o^C for 3 min - 1 repeat.

Cycle 2: 96^o^C for 30 sec, 68^o^C for 1 min (decreasing from 68^o^C to 58^o^C by 0.5^o^C with each repeat), 68^o^C for 2 min - 20 repeats.

Cycle 3: 96^o^C for 30 sec, 55^o^C for 1 min, 68^o^C for 2 min - 15 repeats.

Cycle 4: 68^o^C for 15 min, hold at 10^o^C - 1 repeat.

### Y-SNP and autosomal control primer sequences

#### hu98Y (rs1865680)

Y-rs1865680F1: 5' - ACACTGGGTTCCTTTCAGGGC - 3'

Y-rs1865680R1: 5' - CCCACAACTGAAACTGGCAATG - 3'

#### hu111Y (rs2032624)

Y- rs2032624F1: 5' - TGTTGAACTGAAAGTTGATGCCAC - 3'

Y- rs2032624R1: 5' - CAGCCTTCAAAGCTTCTCCTGG - 3'

#### hu209Y (rs17307398)

Y-rs17307398F1: 5' - TGGGGGTGAATTCAGATTCTCTG - 3'

Y-rs17307398R1: 5' - ACAATGAAATTGAGAAGCTGTCAAGC - 3'

Y-rs17307398R2: 5' - AGCAATCTGAGTGATCCTCAATCTAAACT - 3'

F+R1 primers amplified a 250 bp fragment, while F+R2 primers amplified a 377 bp fragment. Both fragments were amplified, as shown in Supplementary Figure S2.

#### ABO Exon 6, blood type O

ABO_exon6F: 5' - GCAGAAGCTGAGTGGAGTTT - 3'

ABO_exon6R: 5' - TAACCCAATGGTGGTGTTCTG - 3'

#### TP53 Intron 1 VNTR

p53_VNTRF7: 5' - CTTTCCTCAACTCTACATTTCCCATAATACATAGA - 3'

p53_VNTRR1: 5' - ACAAAACATCCCCTACCAAACAGC - 3'

The size of the VNTR amplicon varies with the number of pentanucleotide repeats present in the target TP53 intron 1 sequence. It is 423 bp for M14, ML14, and MDA-MB-435 due to having 9 repeats of A_4_T.

#### hu9 (rs560681)

hu9-rs560681F: 5' - GGGAAACACTGTCATGTACACATGGACC - 3'

hu9-rs560681R: 5' - TTAGCATCGGAATCGAGACGTGC - 3'

#### hu47 (rs447818)

hu47-rs447818F: 5' - ACAGGATCTGAAGCTCTGTGTACTGG - 3'

hu47-rs447818R: 5' - GGAGTGCCAAGGAACATGGTAAGAGC - 3'

# Supplementary References

(1) Butler JM, Hill CR, Coble MD. Variability of new STR loci and kits in US population groups. [www.promega.com.au/resources/profiles-in-dna/2012/variability-of-new-str-loci-and-kits-in-us-population-groups/](http://www.promega.com.au/resources/profiles-in-dna/2012/variability-of-new-str-loci-and-kits-in-us-population-groups/) 2012.

(2) Emanuel SL, Pestka S. Amplification of specific gene products from human serum. Genet Anal Tech Appl 1993; 10: 144-6.

(3) Jen J, Wu L, Sidransky D. An overview on the isolation and analysis of circulating tumor DNA in plasma and serum. Ann N Y Acad Sci 2000; 906: 8-12.

(4) Capes-Davis A, Reid YA, Kline MC, Storts DR, Strauss E, Dirks WG, Drexler HG, Macleod RA, Sykes G, Kohara A, Nakamura Y, Elmore E, Nims RW, Alston-Roberts C, Barallon R, Los GV, Nardone RM, Price PJ, Steuer A, Thomson J, Masters JR, Kerrigan L. Match criteria for human cell line authentication: Where do we draw the line? Int J Cancer 2012; 132: 2510-9.

(5) Yu M, Selvaraj SK, Liang-Chu MM, Aghajani S, Busse M, Yuan J, Lee G, Peale F, Klijn C, Bourgon R, Kaminker JS, Neve RM. A resource for cell line authentication, annotation and quality control. Nature 2015; 520: 307-11.

(6) Edelmann J, Szibor R. Validation of the HumDXS6807 short tandem repeat polymorphism for forensic application. Electrophoresis 1999; 20: 2844-6.

(7) Turrina S, Atzei R, Filippini G, De Leo D. Development and forensic validation of a new multiplex PCR assay with 12 X-chromosomal short tandem repeats. Forensic Sci Int Genet 2007; 1: 201-4.

(8) Hearne CM, Todd JA. Tetranucleotide repeat polymorphism at the HPRT locus. Nucleic Acids Res 1991; 19: 5450.

(9) Edelmann J, Deichsel D, Hering S, Plate I, Szibor R. Sequence variation and allele nomenclature for the X-linked STRs DXS9895, DXS8378, DXS7132, DXS6800, DXS7133, GATA172D05, DXS7423 and DXS8377. Forensic Sci Int 2002; 129: 99-103.

(10) Szibor R, Edelmann J, Zarrabeitia MT, Riancho JA Sequence structure and population data of the X-linked markers DXS7423 and DXS8377: clarification of conflicting statements published by two working groups. Forensic Sci Int 2003; 134: 72-3.

(11) Liang-Chu MM, Yu M, Haverty PM, Koeman J, Ziegle J, Lee M, Bourgon R, Neve RM Human biosample authentication using the high-throughput, cost-effective SNPtrace(TM) system. PLoS One 2015; 10: e0116218.
